# Supramolecular complexation of C_60_ and C_70_ by helical nanographene incorporating *N*-heterotriangulene and hexabenzocoronene subunits†

**DOI:** 10.1039/d4ra07837c

**Published:** 2025-01-31

**Authors:** Marina Kinzelmann, Nina Fröhlich, Frederik Gnannt, Jan Borstelmann, Stefan Frühwald, Christoph Oleszak, Norbert Jux, Andreas Görling, Milan Kivala, Thomas Drewello

**Affiliations:** a Physical Chemistry I, Friedrich-Alexander-Universität Erlangen-Nürnberg Egerlandstraße 3 91058 Erlangen Germany thomas.drewello@fau.de; b Organic Chemistry I, Friedrich-Alexander-Universität Erlangen-Nürnberg Nikolaus-Fiebiger-Straße 10 91058 Erlangen Germany; c Theoretical Chemistry, Friedrich-Alexander-Universität Erlangen-Nürnberg Egerlandstraße 3 91058 Erlangen Germany; d Erlangen National High Performance Computing Center (NHR@FAU) Martensstr. 1 91058 Erlangen Germany; e Organic Chemistry II, Friedrich-Alexander-Universität Erlangen-Nürnberg Nikolaus-Fiebiger-Straße 10 91058 Erlangen Germany; f Institute of Organic Chemistry, Ruprecht-Karls-Universität Heidelberg Im Neuenheimer Feld 270 69120 Heidelberg Germany

## Abstract

Supramolecular host–guest complexes are studied in the gas-phase evaluating a new host molecule for fullerenes (C_60_ and C_70_). The new host molecule is a double *N*-heterotriangulene-[5]helicene (NTH), consisting of two *N*-heterotriangulene (*N*-HTA) blades embedded into a hexabenzocoronene-like backbone with helically curved topology. Host–guest complexes of [1:1]^+^˙^/2+^, [1:2]^+^˙^/2+^, [2:1]^2+^ and [2:3]^2+^ stoichiometry and charge state are formed by electrospray ionization-mass spectrometry (ESI-MS). Ion formation occurs through electrochemical oxidation of the *N*-HTA moieties. Energy-resolved collision-induced dissociation (ER-CID) experiments reveal the noncovalent binding of the fullerenes to the NTH molecule and provide an order of stability for the complexes. Density-functional theory (DFT) calculations establish the lowest energy geometries of the complexes.

## Abstract 2

The host–guest chemistry of the double *N*-heterotriangulene-[5]helicene and C_60,70_ is reminiscent of a molecular helter skelter. The noncovalent nanographene–fullerene complexes are prime examples of concave–convex shape complementarity.

## Graphical abstract

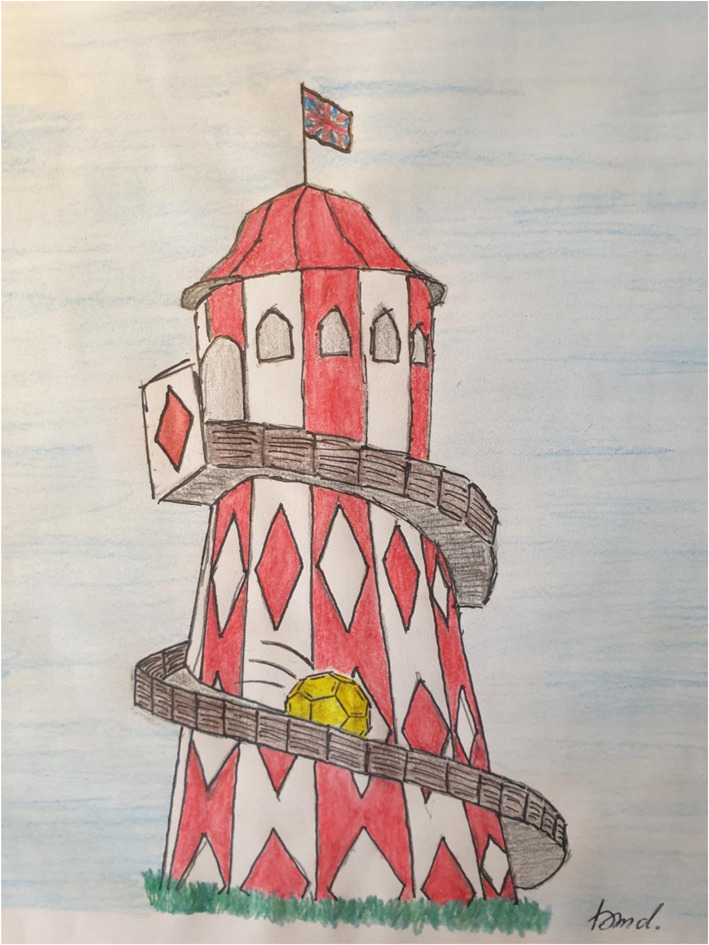

## Introduction

In recent years, tremendous progress has been made in the area of molecular nanographenes.^1–26^ Synthetic efforts were directed towards bottom-up approaches in order to gain control over size, structure and properties of the nanographenes.^27,28^ A major motivation behind these efforts lies in their potential applications in photovoltaic, molecular electronics and sensing.^2,29–32^ Nanographenes are particularly suited to overcome the zero band gap problem^2^ of pristine single-layer graphene, which poses severe limitations on electronic applications of graphene.^33^ Crucial to improving the applicability of molecular nanographenes is the detailed understanding of electron and energy transfer processes^34^ and connected with this, insight into the interaction of nanographenes with other molecules. Along those lines, the host–guest chemistry of nanomaterials with fullerenes (C_60_ and C_70_) has been of prime interest. Fullerenes are widely established as electron acceptors in electronic applications.^35^ But also their unique spherical structure has an impact on their host–guest chemistry. The importance of shape complementarity to the host–guest chemistry of nanomaterials with fullerenes has been the subject of several reviews.^36–39^ Crucial to the formation and stability of such complexes is thus the precise fit of host and guest molecule. Noncovalent interaction and supramolecular complex formation with the convex fullerene surface will be enhanced through a complementary concave surface of the nanographene. In other words, for the perfect fit with the fullerene, the nanomaterial should be curved. Curvature can be induced into the two-dimensional (2D) honey-comb lattice by introducing non-hexagonal rings.^40^ The implementation of a five-membered ring (or lower) will lead to positive Gaussian curvature (bowl shape structure) and introducing a seven-membered ring (or higher)^41,42^ results in negative Gaussian curvature (saddle shape structure). There are even examples of nanographenes possessing both curvature motifs simultaneously.^43–48^ Retaining the six-membered rings, curvature can be created by introducing a helical twist to the molecule.^40^

**Fig. 1:**
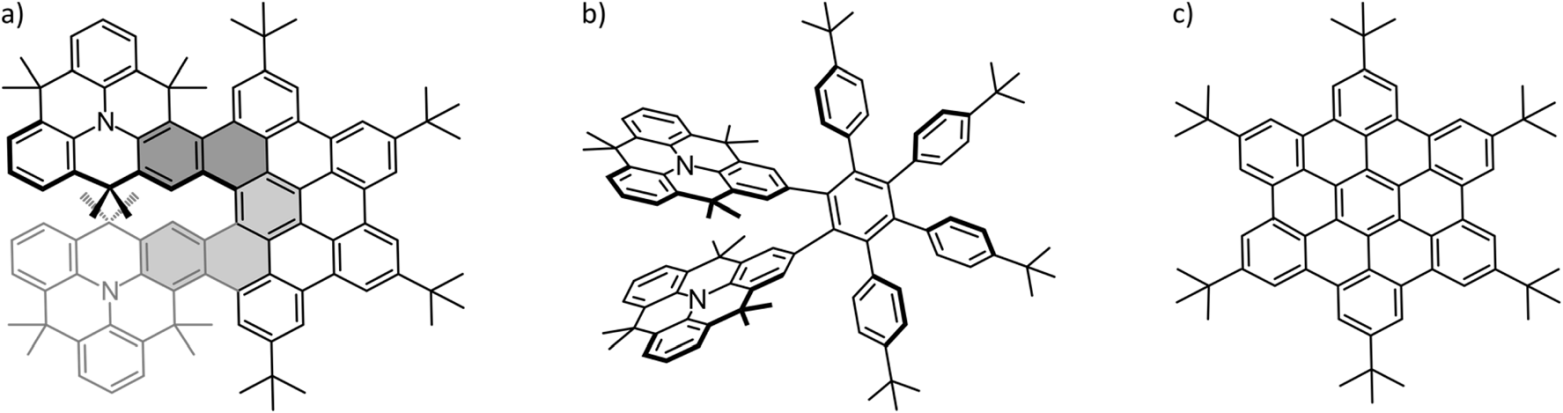
Structure of the investigated molecules: (a) NTH, (b) the NTH precursor and (c) ^*t*^butyl-decorated hexabenzocoronene (HB-HBC).

A helical twist can be imposed on a system by intentionally inducing steric repulsion between different, overlapping parts of the molecule. This is the case in the title molecule of this study shown in Fig. 1a. In the double *N*-heterotriangulene-[5]helicene (NTH) studied here, two *N*-heterotriangulene (*N*-HTA) units constitute the terminal parts of a [5]helicene. The [5]helicene is part of a π-extension which is reminiscent of the hexa-*peri*-hexabenzocoronene (HBC) molecule (Fig. 1c). The dimethylmethylene bridges of the *N*-HTA moieties protrude out of the molecular plane, causing steric repulsion of the two units, ultimately leading to a distortion of the π-system from planarity. This curvature in the NTH structure offers potentially a good interaction area for the formation of supramolecular complexes with C_60_ and C_70_. Fig. 1 also depicts the synthetic precursor of the NTH molecule (Fig. 1b), which turns into the NTH molecule by cyclodehydrogenation.

The gas-phase host–guest chemistry of single-core *N*-HTA hosts with C_60_ has been the topic of an earlier investigation by us.^49^ In the present study, we employ electrospray ionization-(tandem) mass spectrometry (ESI-MS(/MS)) to ionize the NTH molecule and characterise the complexes formed with C_60_ and C_70_. ESI is a soft ionization method, which may allow both the direct transfer of intact noncovalent complexes from solution into the gas-phase, as well as their formation through aggregation phenomena during the spraying process. The mass spectrometry-based gas-phase experiment on solitary ions enables the investigation of intrinsic properties within complexes without solvent effects or the influence of counter ions.^50^ Hosseini *et al.* showed that the fullerene solubility has an inverse correlation on the binding strength of host–guest complexes.^51^ Therefore, association constants measured in solution will be dependent upon the employed solvent. Energy-resolved collision-induced dissociation experiments provide insight into the fragmentation dynamics of noncovalent complexes and allow the establishment of relative stability orders. Thus, gas-phase studies of host–guest systems provide insight into intermolecular interactions, representing a unique complementary to condensed phase or solid state investigations. DFT calculations accompany the experimental findings and are used to establish the geometries of the complexes.

## Experimental

The racemic NTH was synthesized following a reported procedure.^52^ A dedicated publication detailing the NTH synthesis and its photophysical and redox properties will be published separately.^53^ The ^1^H- and ^13^C-NMR spectra of NTH, as well as a link to the crystal structure are provided in the ESI.† The fullerenes (C_60_ and C_70_) and trifluoroacetic acid (TFA) were purchased from Merck. The solvents dichloromethane (DCM), acetonitrile (ACN) and toluene were purchased from VWR chemicals in HPLC grade purity. The stock solutions of NTH and the fullerenes were prepared in DCM (0.5 g L^−1^) and toluene (1.0 g L^−1^), respectively. The final concentration for the ESI measurements were 1 × 10^−5^ M and 5 × 10^−5^ M for the NTH and fullerenes, respectively, in a mixture of DCM and ACN (1 : 1; v : v) to which a small amount of TFA (2 μL) was added.

All MS^1^ and MS^2^ measurements were performed with a quadrupole time-of-flight (qToF) instrument (micrOTOF-QII, Bruker Daltonics, Bremen, Germany). The analyte solution was directly injected into the ESI source with a flow rate of 3.0 μL min^−1^; the temperature of the nitrogen counter flow was set to 180 °C. All measurements were performed in the positive ion mode, whereby the capillary voltage was set to 4.5 kV and the endplate offset to −0.5 kV. For the MS^2^ experiments, the precursor ions were selected by the mass analyzer quadrupole and accelerated into the collision cell quadrupole. A Parker LCMS64 nitrogen generator provided the nitrogen, used as a collision gas, with a purity of 99.999% and a flow rate of 0.5 L min^−1^. The instrument parameters were optimized to obtain good intensities for each experiment.

The survival yield (SY)^54,55^ of the precursor ions was calculated as the intensity ratio of the ion of interest to all observed ions and was recorded as a function of the collision energy in the centre-of-mass frame (*E*_com_).
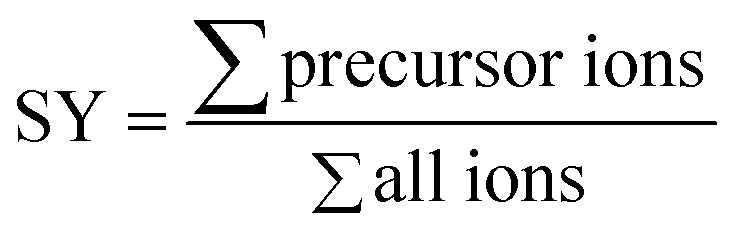
*E*_com_ is derived from the laboratory frame collision energy (*E*_lab_), the molecular mass of the collision gas (*M*_N_2__) and of the investigated precursor ion (*M*_ion_).
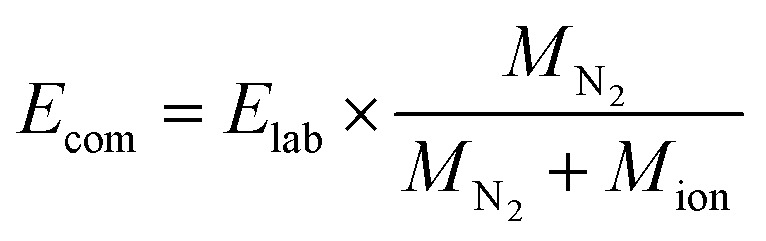


The breakdown graphs were obtained under multiple collision conditions and fitted with a sigmoid function. The collision energy, *E*_50_, at witch 50% of the precursor ions is dissociated is used as a measure of its relative stability.

DFT calculations on the formed complexes were performed with the TURBOMOLE program package (Version 7.3).^56^ Because of the increased size of the fullerene aggregates the GGA functional PBE was used in combination with the basis set def2-TZVP^57^ and the D3 dispersion correction to correctly account for the dispersion interactions between NTH and fullerene.^58^ Fragmentation energies were computed using fully relaxed structures of the complexes and fragments, respectively. An estimation of the BSSE error can be found in the ESI.† For the visualization of these interactions the program NCIPLOT (Version 3.0) was used.^59^ Areas of noncovalent interactions were determined by calculating and visualizing contour plots of the reduced density gradient given by
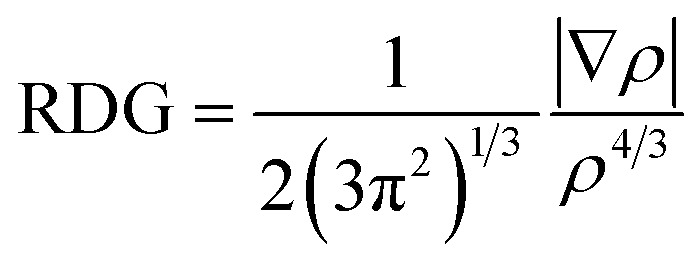
with the electron density *ρ*.

The contour plots were visualized at areas of low electron density below a chosen threshold, for which *ρ* = 0.2 was selected. In order to identify the attractive or repulsive nature of the noncovalent interaction the expression sign(*λ*_2_)*ρ* was evaluated, which contains the electron density *ρ* and the second largest eigenvalue of the electron density Hessian matrix which changes its sign according to the nature of the noncovalent interaction. For the visualization isosurfaces of the RDG at 0.3 arbitrary units were used and colored according to a color scale of −0.05 < *ρ* < 0.05.

## Results and discussion

**Fig. 2:**
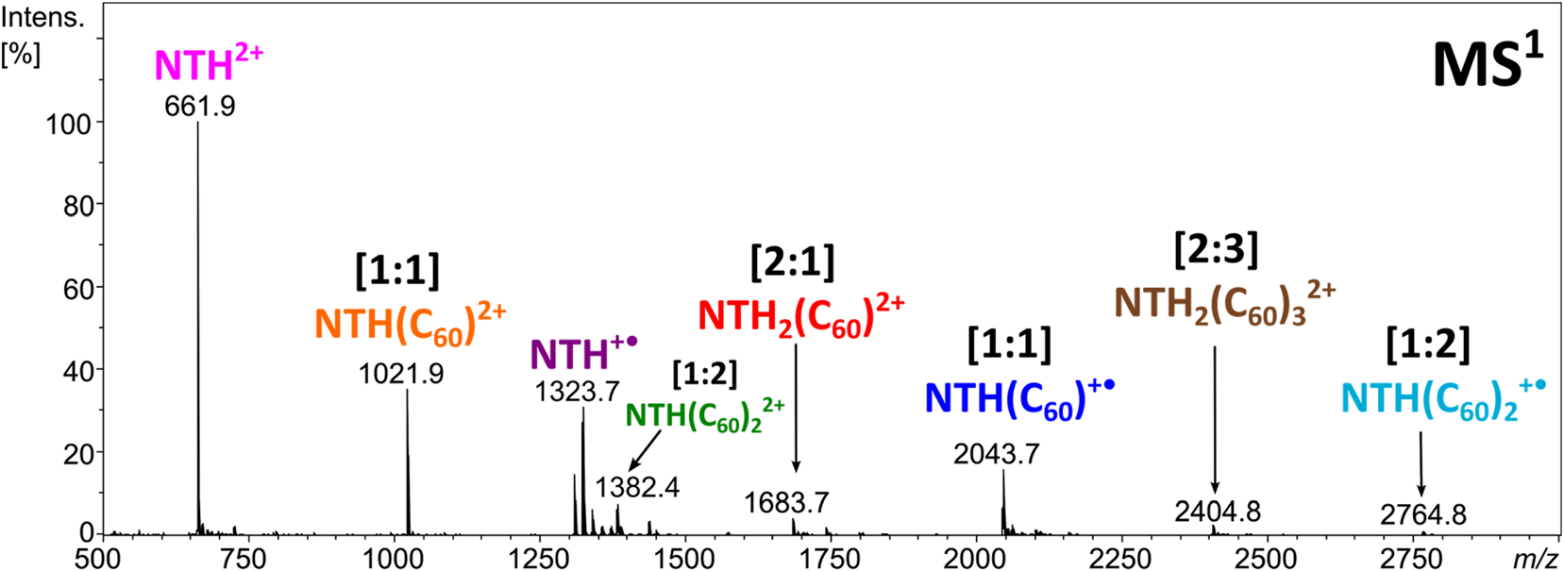
Positive ion-mode ESI-MS of the NTH/C_60_ analyte solution.

Electrospraying a NTH/C_60_ analyte solution results in the positive-ion ESI mass spectrum displayed in Fig. 2. The most abundant ion corresponds to the molecular dication NTH^2+^ (*m*/*z* 661.9). The other intense signals are assigned to the molecular ion NTH^+^˙ (*m*/*z* 1323.7) as well as to the doubly and singly charged [1:1] NTH–fullerene complexes at *m*/*z* 1021.9 and 2043.7, respectively. Less intense are the signals for the [1:2] NTH(C_60_)_2_^+^˙, [1:2] NTH(C_60_)_2_^2+^, [2:1] NTH_2_(C_60_)^2+^ and the [2:3] NTH_2_(C_60_)_3_^2+^ complexes at *m*/*z* 2764.8, *m*/*z* 1382.4, *m*/*z* 1683.7 and *m*/*z* 2404.8, respectively. The molecular ions of the bare NTH host are the most abundant species observed, followed by the [1:1] complexes with C_60_ and eventually the larger complexes with more than only one host and/or guest molecule incorporated. This product distribution is fully in line with a plausible formation scenario in which the [1:1] complex is initially formed with further coordination to it by NTH and/or C_60_ towards the larger complexes. All ions observed are either radical cations or dications and as such the result of single or double electron transfer reactions. Considering that the ion formation by ESI is commonly based on acid/base chemistry, the essential lack of protonation is truly remarkable. However, the trialkylamine building block as being present in the *N*-HTA units is amendable to facile electrochemical oxidation. Redox reactions may occur as intrinsic electrochemical processes within the ESI source.^60–64^ Previous ESI studies on azatriangulenes confirmed their facile electrochemical oxidation.^49,54,65^ Therefore, one can confidently conclude that oxidation occurs at the *N*-HTA unit(s). This means for the complexes observed in Fig. 2 that the NTH molecule carries the charge and C_60_ is present as a neutral molecule.^49^ This conclusion is also corroborated by the results of the dissociation experiments of these complexes (*vide infra*).

Clearly, the MS^1^ experiment provides evidence of the formation of multiple host–guest complexes with NTH and C_60_, featuring the prominent formation of singly and doubly ionised [1:1] complexes and – to a lesser extent also – larger complexes. The question arises as to whether or not the observed aggregation extent is indicative of a particularly favourable interaction within the host–guest complex or if similar results would also be obtained with other host molecules of comparable size but different structure. Moreover, due to the helical structure of the NTH molecule, there are two potential binding sites for complex formation. This refers to either interaction of the guest with both of the *N*-HTA units in a tweezer-like fashion. On the other hand, the fullerene could interact with the backbone of the molecule. In order to answer these questions, we performed experiments with two different host molecules containing the relevant structural elements and allowing decisive insight.

Firstly, we applied the synthetic uncyclized precursor of NTH as a host (Fig. 1b), for which the resulting mass spectrum shows no complex formation with C_60_ at all. The NTH precursor still features the tweezer-like arrangement of the two *N*-HTA moieties, although the cyclodehydrogenation to form the HBC-backbone has not yet occurred. Instead, the phenyl groups are arranged in a propeller-shaped fashion to avoid steric repulsion (see Fig. S2†). The cavity between the two *N*-HTA moieties is thus available in both hosts, NTH (Fig. 1a) and its precursor (Fig. 1b) and constitutes a highly attractive binding site for metal cations. Such a tweezer-like binding site is well established for all-carbon helicenes.^66^ The fact that the NTH precursor shows no complexation with C_60_ has a structural implication for the observed [1:1] complex of C_60_ with NTH. As a matter of fact, we assume that the cavity of the *N*-HTA tweezer is not well suited to form stable complexes with the fullerene, simply because the fullerene is too big for a favourable interaction. This should be even more the case for the NTH host where movements are more restricted because of the ridged backbone. Thus, the tweezer binding motif is not operative in this system. A bigger host system based on a porphyrin-decorated HBC has been able to effectively attach up to eight C_60_ molecules.^67^ As a conclusion, in the observed complexes with NTH, the C_60_ must be associated with the “outside” of the NTH host.

Secondly, since the HBC-backbone appears to be an essential requirement for the complexation, we tested hexa-*tert*-butyl-hexa-*peri*-hexabenzocoronene (HB-HBC) as a host (Fig. 1c). The resulting mass spectra (Fig. S3†) are characterized by intense signals of mono- and dimeric structures of the HB-HBC molecule. The mass spectra reveal the formation of only very minor amounts of an HB-HBC fullerene complex of [2:1] composition. CID experiments show that the fullerene is only loosely bound to an HB-HBC dimeric structure (Fig. S4†). The complex dissociates into C_60_ and the dimer cation of HB-HBC and this occurs at much lower collision energies than the dissociation of the [1:1] complex of C_60_ with NTH^+^˙. The signal of a [1:1] C_60_:HB-HBC complex can only just be identified, but is too weak to be examined further. The low abundance of the [1:1] and [2:1] complexes, together with the low bond strength between C_60_ and HB-HBC dimer ion, indicate that complex formation is possible but not efficient. This can be attributed to a poor shape-complementarity of the planar HB-HBC and the convex surface of the fullerene.^26^

The experiments with the three different host molecules indicate that the observed extent of complexation of NTH with C_60_ is significant and that the curved structure of the HBC-backbone is clearly the decisive factor for the successful complex formation with C_60_. Interaction of the fullerene with the curved HBC-backbone, perhaps even including one of the *N*-HTA units, would result in a convex–concave binding motif, for which many examples are reported in the literature.^36–39^ This proposed binding motif will be reconsidered in the discussion of the DFT calculations (*vide infra*).

**Fig. 3:**
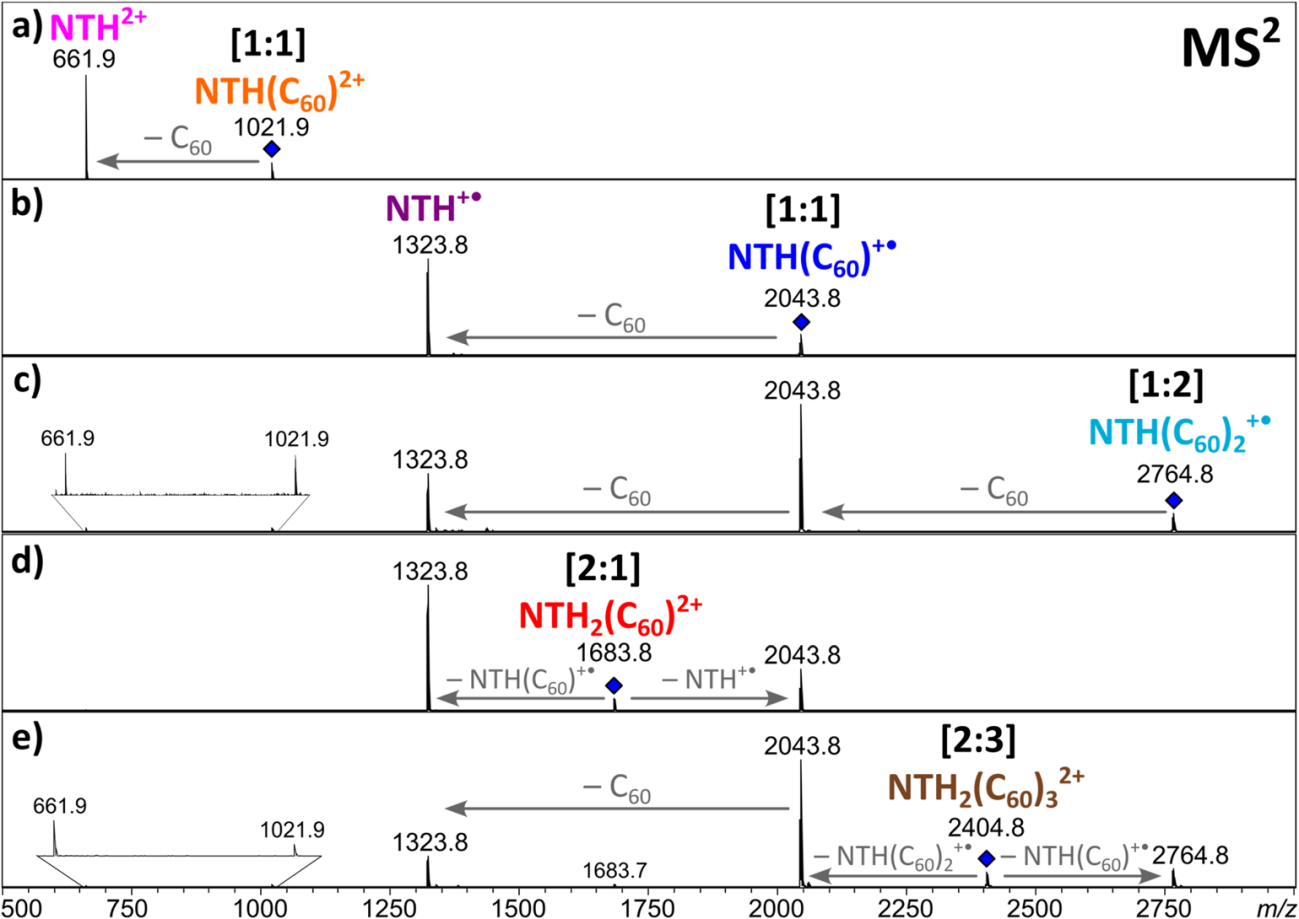
MS^2^ spectra of the selected NTH/C_60_ complexes.

In order to obtain further insight into the composition of the complexes and the charge distribution within the complexes, collision-induced dissociation (CID) of the ions was studied in MS^2^ experiments. The singly and doubly charged [1:1] complexes both fragment by the loss of a neutral fullerene, leading to NTH^+^˙ and NTH^2+^, respectively (Fig. 3a and b). Also, the [1:2] complex (Fig. 3c) shows the successive release of two C_60_s. The two doubly charged complexes, [2:1]^2+^ (Fig. 3d) and [2:3]^2+^ (Fig. 3e), dissociate by a reaction which is known as Coulomb explosion,^55,68,69^ which refers to the dissociation of a dication into two singly charged fragment ions. NTH_2_(C_60_)^2+^ dissociates into NTH^+^˙ and (C_60_)NTH^+^˙ and NTH_2_(C_60_)_3_^2+^ decomposes into NTH(C_60_)^+^˙ and NTH(C_60_)_2_^+^˙. These reactions suggest that the two positive charges are located on each of the two NTH molecules in the complexes. There is no indication of a reaction into a smaller fragment complex with only one doubly charged NTH unit which would have supported the alternative initial charge distribution of one NTH^2+^ dication and one neutral NTH molecule in the complex. All dissociation reactions confirm that NTH was charged while C_60_ is the neutral component in the complex.

**Fig. 4:**
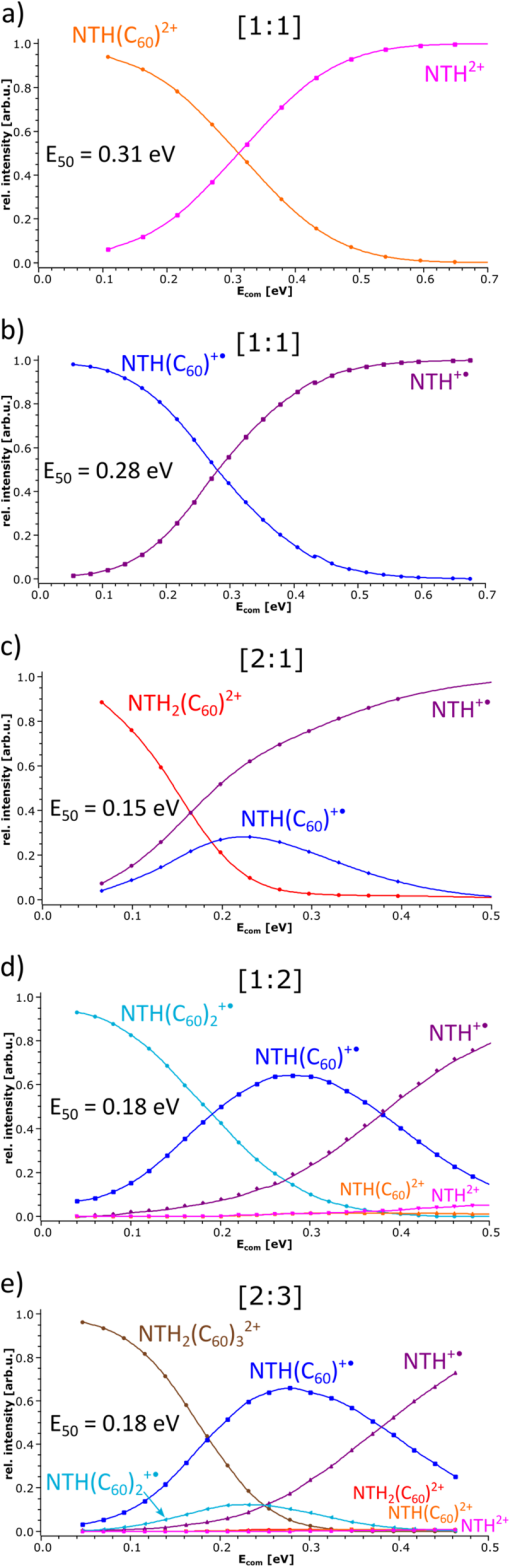
Breakdown graphs of the selected NTH/C_60_ complexes.

In the following, the stability of the complexes is evaluated by energy-dependent CID measurements. In these experiments, a selected complex is collisionally activated to induce decomposition. By increasing the collision energy, a breakdown graph is obtained. The *E*_50_ value represents the collision energy at which half of the population of the selected complex is decomposed and is taken as a measure of stability. By comparing the breakdown graphs of the singly and doubly charged [1:1] complexes (Fig. 4a and b), one notices that the doubly charged complex decomposes at a slightly higher collision energy. NTH(C_60_)^2+^ has an *E*_50_ value of 0.31 eV, and NTH(C_60_)^+^˙ possesses an *E*_50_ value of 0.28 eV. The dicationic complex is thus more stable than the singly charged complex. At first sight this finding appears somewhat puzzling since charge repulsion should weaken the dication and allow for more facile dissociation.^70^ However, in the host–guest complex, the Coulomb repulsion within the NTH unit does not affect the complex stability, as the charge repulsion is only restricted to the host entity and does not affect the guest molecule. In contrast, the two charges may even enhance the polarization of the C_60_ guest and lead to a more firmly connected complex. Our DFT calculations (*vide infra*) also confirm that the dissociation of dicationic NTH(C_60_)^2+^ into NTH^2+^ and neutral C_60_ (Fig. 3a) requires more energy than the fragmentation of the monocationic NTH(C_60_)^+^˙ into NTH^+^˙ and neutral C_60_ (Fig. 3b).

The breakdown graphs of the larger complexes beyond the [1:1] composition (Fig. 4c–e) reveal a clearly reduced complex stability. The larger complexes possess approximately only half of the stability seen for the [1:1] complexes. The *E*_50_ values were obtained as 0.15 eV for NTH_2_(C_60_)^2+^ (Fig. 4c), 0.18 eV for NTH(C_60_)_2_^+^˙ (Fig. 4d) and again 0.18 eV for the largest complex NTH_2_(C_60_)_3_^2+^ (Fig. 4e). The lower stability confirms the assumption that larger entities are generated by the addition of further building blocks to the initial [1:1] complex. Amongst the larger complexes, NTH_2_(C_60_)^2+^ (Fig. 4c) is by a small margin the weakest complex. This may be caused by a somewhat more pronounced repulsion of the two positive charges in this complex. In NTH_2_(C_60_)^2+^ (Fig. 4c) there is only one C_60_ to accommodate the two positively charged NTH^+^˙ cations, while in NTH_2_(C_60_)_3_^2+^ (Fig. 4e) two more C_60_s may stabilise the complex. The other larger complex NTH(C_60_)_2_^+^˙ (Fig. 4d) is just singly charged and therefore not affected by Coulombic repulsion. The breakdown graphs also reveal the fragmentation dynamics of the larger complexes. NTH_2_(C_60_)^2+^ (Fig. 4c) with two NTH^+^˙ cations attached to one neutral C_60_ decomposes almost directly into NTH^+^˙ and the intermediate NTH(C_60_)^+^˙ is not as abundantly formed as for the other larger complexes. On the one hand, NTH^+^˙ is already formed in the Coulomb explosion of NTH_2_(C_60_)^2+^ into NTH^+^˙ and NTH(C_60_) ^+^˙. But also, the resulting NTH(C_60_)^+^˙ seems to dissociate much more easily than in Fig. 4d when it is formed from NTH(C_60_)_2_^+^˙ by a simple loss of neutral C_60_. We assume that the Coulomb explosion reaction leads to an excitation of the NTH(C_60_)^+^˙ intermediate, which decomposes more easily. NTH(C_60_)_2_^+^˙ (Fig. 4d) shows after the loss of one C_60_ the pronounced formation of an intermediate [1:1] NTH(C_60_)^+^˙ complex, which eventually undergoes a second C_60_ loss to result in the formation of bare NTH^+^˙. Despite its different composition, NTH_2_(C_60_)_3_^2+^ (Fig. 4e) shows almost exactly the same breakdown behaviour as NTH(C_60_)_2_^+^˙ (Fig. 4d). This is caused by the fact that NTH_2_(C_60_)_3_^2+^ (Fig. 4e) decomposes by a Coulomb explosion reaction efficiently into NTH(C_60_)^+^˙, the central [1:1] intermediate fragment ion and into NTH(C_60_)_2_^+^ which also easily decomposes into NTH(C_60_)^+^˙ by C_60_ loss. The breakdown graphs of the NTH(C_60_)_2_^+^ complex (Fig. 4d) and the NTH_2_(C_60_)_3_^2+^ complex (Fig. 4e) show minute signals for the doubly charged ions: NTH^2+^, NTH(C_60_)^2+^ and NTH_2_(C_60_)^2+^ (only observed for NTH_2_(C_60_)_3_^2+^) in the MS^2^ spectra at elevated collision energies (*E*_com_ > 0.32 eV). These doubly charged ion signals appear only after complete decomposition of the [2:3] complex and are therefore not connected to direct dissociations of the selected precursor complex. In fact, a likely source of these unwanted, interfering ions are even larger multiply charged complexes of the same *m*/*z* value (isobaric ions) as the mass-selected ions of interest.

Unfortunately, the [1:2] complex ion NTH(C_60_)_2_^2+^ could not be isolated from a protonated species which interfered with the ion of interest, so that no reliable *E*_50_ value could be obtained. However, the dissociation pattern shows as expected the consecutive loss of two C_60_s into NTH^2+^ as the final ion.

The experiments were also performed with C_70_. Two different binding modes result from the oval shape of C_70_: “end-on” binding, which resembles the binding of C_60_, and “side-on” binding. Which binding motif is preferred depends on the structural arrangement of the molecule. In the absence of steric hindrance, side-on binding is commonly preferred because the interaction area is larger than it is for end-on binding.^71^ Our experiments show (Fig. S5†) that more NTH–fullerene complexes are formed for C_60_ (*e.g.* [2:1] and [2:3]) than for C_70_. Indicating that complex formation with C_60_ is more efficient. However, those C_70_ complexes that were observed are all more stable compared to the same complexes with C_60_ (Fig. S6†). Which clearly indicates that there is more binding interaction with C_70_. The increased complex stability is most evident for the mono and dicationic [1:1] complexes. For the [2:1] complex, only a minor increase of the complex stability could be determined. This agrees with the assumption that the larger complexes are the result of lose coordination of additional NTH molecules or fullerenes to an already existing [1:1] complex. Therefore, we do not expect the same interaction strength as for the [1:1] complexes. A list with all determined *E*_50_ values for comparison of complex stabilities can be found in the ESI (Table S1†).

**Fig. 5:**
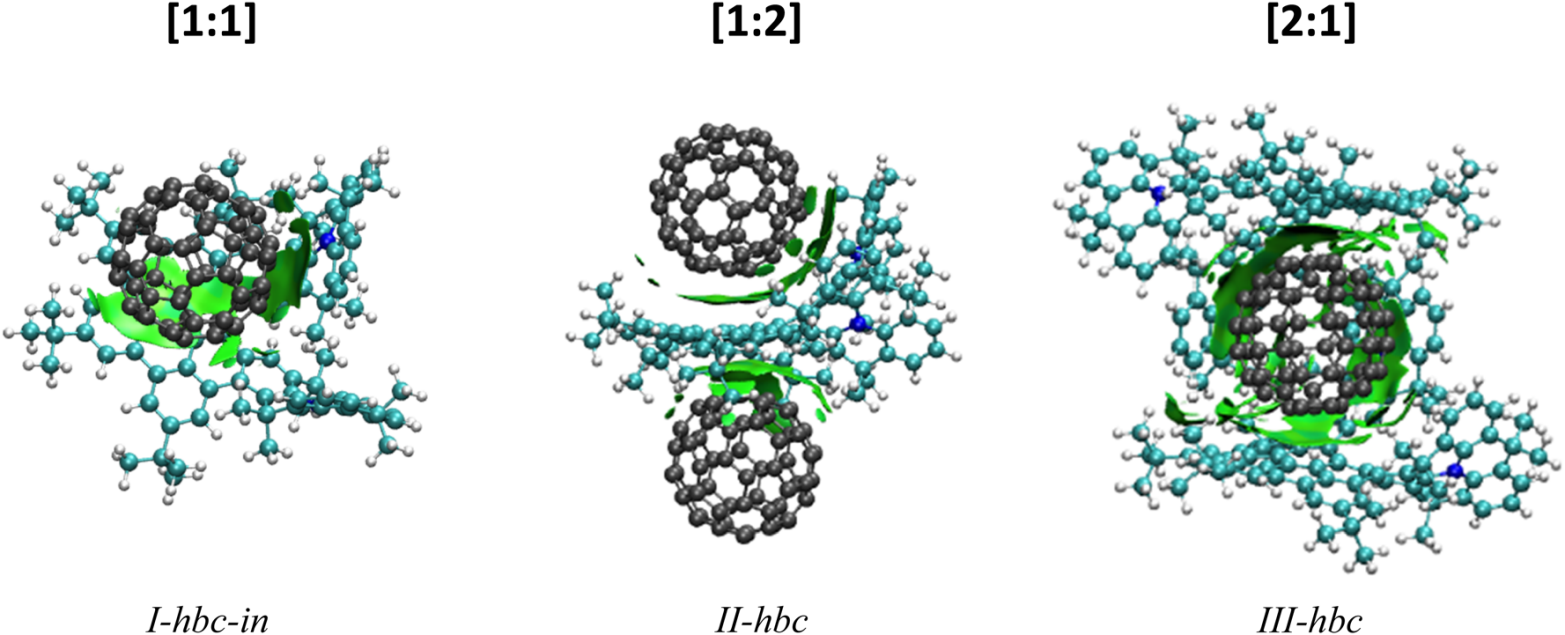
DFT-optimized geometries of the NTH/C_60_ complexes with visualization of long-range interactions.

DFT calculations were performed in support of the experimental findings. The purpose of these calculations is it to identify the lowest energy structures of the host–guest complexes and to establish their fragmentation energies. Due to the large system size, only the [1:1], [1:2] and [2:1] NTH/C_60_ complexes were calculated in the gas phase. The most likely position of the fullerene guest with respect to the NTH host, as well as the energy required for complex dissociation were calculated. Fig. 5 displays the most stable complex geometries. The other structures as well as a table of all fragmentation energies can be found in the ESI (Fig. S8 and Tables S2–S4†). For the [1:1] complex, three complex geometries were considered (see ESI†). The fullerene was either placed between both *N*-HTA units (*I-hta*), between HBC-backbone and the backside of an *N*-HTA unit (*I-hbc-in*) or above the HBC with no further interaction (*I-hbc-out*). The least stable structure could be assigned to the tweezer-like *I-hta* conformation. The *N*-HTA units apparently cannot open wide enough to properly enclose the fullerene. This is in agreement with the experimental results on the basis of which a bonding to the *N*-HTA tweezer could be discounted. The two geometries involving interaction with the HBC backbone form more strongly coordinated complexes. However, the *I-hbc-out* geometry lacks the additional *N*-HTA binding and is, therefore, slightly less stable than the *I-hbc-in* geometry. We therefore assume that the [1:1] complexes observed in the experiments adopt the *I-hbc-in* conformation. For the [1:2] complex, a second fullerene was added to the most stable [1:1] complex. Two structures were considered. The tweezer-like position between the *N*-HTA units (*II-hta*) as well as the position over the other unoccupied side of the HBC-backbone (*II-hbc*). Again, the HBC coordination is favoured, leading to the complex depicted in Fig. 5. For the [2:1] complex, the fullerene was either encapsulated by the *N*-HTA units (*III-hta*) or placed between the HBC-backbones (*III-hbc*) of both NTH molecules. The *III-hbc* position is much more stable, and therefore, we assign this geometry to the [2:1] complex observed in the experiment.

For the three different complex compositions studied here, the fragmentation energies were calculated for the neutral, singly and doubly charged complexes. The fragmentation energy is obtained as the difference in energy of the noncovalent complex to the separated components of the complex (no reverse activation barrier assumed) and – represents the binding energy within the complex. While a list of all fragmentation energies can be found in the ESI (Tables S2–S4†), we will discuss in the following the complexes that were actually observed in the experiments.

On the whole, the fragmentation energies confirm the experimentally observed charge distributions upon dissociation of the complexes. For all singly charged complexes, the charge resides on the NTH rather than on C_60_, which is a consequence of the lower ionization energy of the NTH molecule. This is confirmed even though the ionization energy of C_60_ was calculated as 7.32 eV, which is clearly lower than the well-established experimental value of 7.6 eV,^72,73^ representing a considerable discrepancy. The first and second IE of the NTH molecule were obtained as ^1^IE_NTH_ = 5.33 eV and ^2^IE_NTH_ = 7.49 eV, respectively. There is no experimental gas-phase data available for comparison. For the doubly charged NTH_2_(C_60_)^2+^ complexes with two NTH units the calculations confirm the experimental observation and predict a more favourable charge distribution of NTH^+^˙/NTH^+^˙ rather than NTH^2+^/NTH^0^. For the doubly charged [1:1] complex NTH(C_60_)^2+^ theory predicts the feasibility of electron transfer within the complex from C_60_ (IP = 7.32 eV) to NTH^2+^ (^2^IE_NTH_ = 7.49 eV). Therefore, the fragmentation into C_60_^+^˙ and NTH^+^˙ (fragmentation energy = 1.05 eV, ESI Table S2†) should be favoured over the experimentally observed dissociation into a neutral fullerene and the doubly charged molecule NTH^2+^ (fragmentation energy = 1.22 eV, ESI, Table S2†). Even when C_60_ is replaced by C_70_, no indication of this reaction could be observed. C_70_ has a more favourable thermochemistry towards electron transfer to NTH^2+^, because of its slightly lower IP of 7.47 eV.^74^ Only when C_60_ was replaced by C_78_ in the complex, which possesses an experimental IP of 7.0 eV,^75^ that is 0.6 eV lower than the IP of C_60_, the electron transfer followed by dissociation into NTH^+^˙ and C_78_^+^˙ was abundantly observed. However, the formation of NTH^2+^ and C_78_ was still the more abundantly occurring dissociation reaction (see Fig. S7†). Unfortunately, it is not clear at this point as to whether the thermochemistry of the charge transfer within the NTH^2+^(fullerene) complexes is adequately described at this level of theory. It is also possible that the collision experiment is affected by a kinetic shift. In which case the electron transfer within the complex would not take place if the difference of the second IP of the NTH and the first IP of the fullerene is not sufficiently large.

## Conclusion

The host–guest chemistry of the helical NTH molecule comprising *N*-HTA and HBC moieties with the fullerenes C_60_ and C_70_ has been explored in gas-phase experiments accompanied by DFT analysis of the geometries and fragmentation energies. The NTH molecule is identified as a new host for the fullerenes. The concave–convex shape complementarity is established as the essential prerequisite for complex formation between the helical nanographene host and the spherical fullerene guest. Convex C_60_ binds to the concave helicene-like backbone of NTH, which also involves the terminal *N*-HTA moiety. Since the respective binding site is present twice in both the host and the guest molecule, complex formation beyond the [1:1] stoichiometry occurs leading to [2:1], [1:2] and even bigger complexes. However, complexes beyond the [1:1] composition show less stability. Unfavorable shape matching may prevent the efficient formation of larger complexes with C_70_. However, the larger surface area of C_70_ promotes the development of dispersion forces between NTH and fullerenes, which explains the higher stability of the [1:1] complexes with C_70_ compared to those with C_60_. The findings of this investigation contribute to a better understanding of the noncovalent bonding within complexes of NTH and fullerenes and may aid efforts to enhance the applicability of nanographenes in areas such as photovoltaic, molecular electronics and sensing.

## Data availability

The data supporting this article have been included as part of the ESI.†

## Conflicts of interest

There are no conflicts of interests to declare.

## Supplementary Material

RA-015-D4RA07837C-s001
