# Supplementary material for: Supramolecular complexation of C60 and C70 by helical nanographene incorporating N-heterotriangulene and hexabenzocoronene subunits
Source: RSC Adv. 2025 Jan 31;15(5):3250–8. doi: 10.1039/d4ra07837c (PMC11784497; doi:10.1039/d4ra07837c)
Supplement: RA-015-D4RA07837C-s001 [file RA-015-D4RA07837C-s001.pdf]

Supplementary Information (SI) for RSC Advances.
This journal is © The Royal Society of Chemistry 2025

Supramolecular complexation of C_{60} and C_{70} by helical nanographene incorporating N-heterotriangulene and hexabenzocoronene subunits

Marina Kinzelmann [a)], Nina Fröhlich [b)], Frederik Gnannt [b)], Jan Borstelmann [f)], Stefan Frühwald [c)], Christoph Oleszak [e)], Norbert Jux [e)], Andreas Görling [c,d)], Milan Kivala [f)] and Thomas Drewello [*,a)]

a) Physical Chemistry I, Friedrich-Alexander-Universität Erlangen-Nürnberg, Egerlandstraße 3, 91058 Erlangen, Germany.

b) Organic Chemistry I, Friedrich-Alexander-Universität Erlangen-Nürnberg, Nikolaus-Fiebiger-Straße 10, 91058 Erlangen, Germany.

c) Theoretical Chemistry, Friedrich-Alexander-Universität Erlangen-Nürnberg, Egerlandstraße 3, 91058 Erlangen, Germany.

d) Erlangen National High Performance Computing Center (NHR@FAU), Martensstr. 1, 91058 Erlangen, Germany

e) Organic Chemistry II, Friedrich-Alexander-Universität Erlangen-Nürnberg, Nikolaus-Fiebiger-Straße 10, 91058 Erlangen, Germany.

f) Institute of Organic Chemistry, Ruprecht-Karls-University Heidelberg, Im Neuenheimer Feld 270, 69120 Heidelberg, Germany.

* corresponding author: E-mail: thomas.drewello@fau.de

Contents

1. Reference MS^1 Spectra 2
2. Breakdown graph of $(HB\text{-}HBC)_2(C_{60})^+$ 3
3. MS^1 Spectra of NTH and C_{70} 4
4. Breakdown graphs of NTH(C_{70}) 5
5. MS^2 Spectra of $NTH(C_{78})^{2+}$ 6
6. E_{50} values of the NTH complexes with C_{60} and C_{70} 6
7. DFT calculations: complex structures 7
8. Calculated fragmentation and ionization energies 8
9. Energies and cartesian coordinates of the host-guest complexes 9
10. ^1H-, ^{13}C-NMR spectra and crystal structure of NTH 9

1. Reference MS^1 Spectra

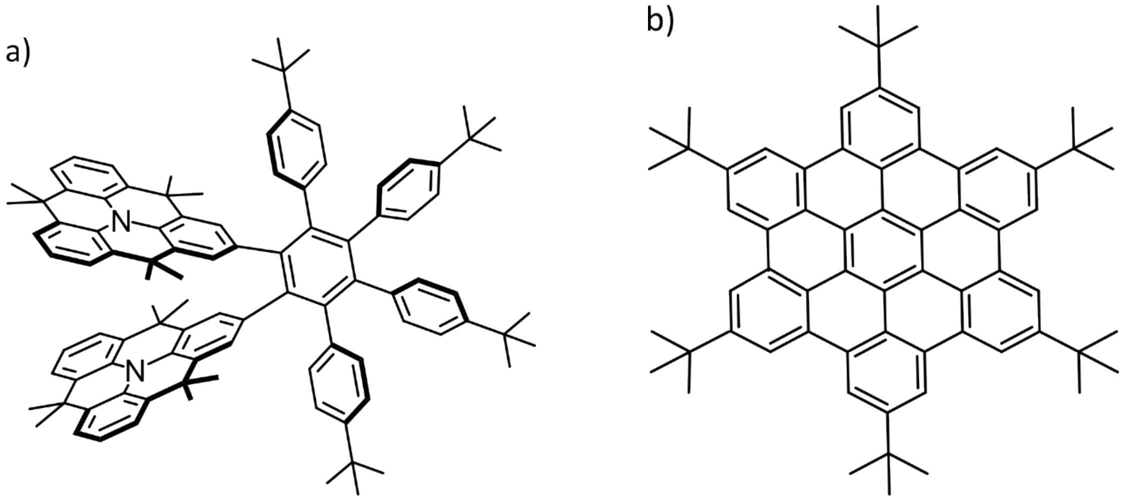

Figure S 1: Reference molecules for comparison with NTH. a) NTH precursor (M), b): hexa-tert-butyl-hexa-peri-hexabenzocoronene (HB-HBC)

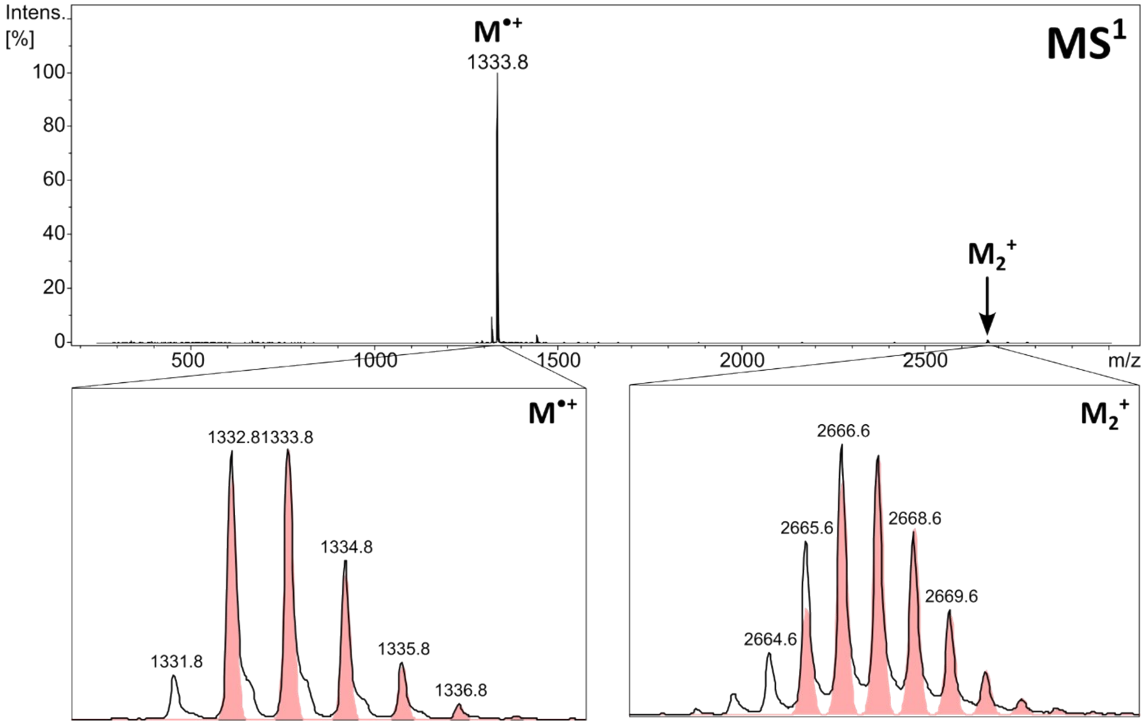

Figure S 2: MS^1 of the NTH precursor/C_{60} analyte solution. No molecule-fullerene complexes could be observed.

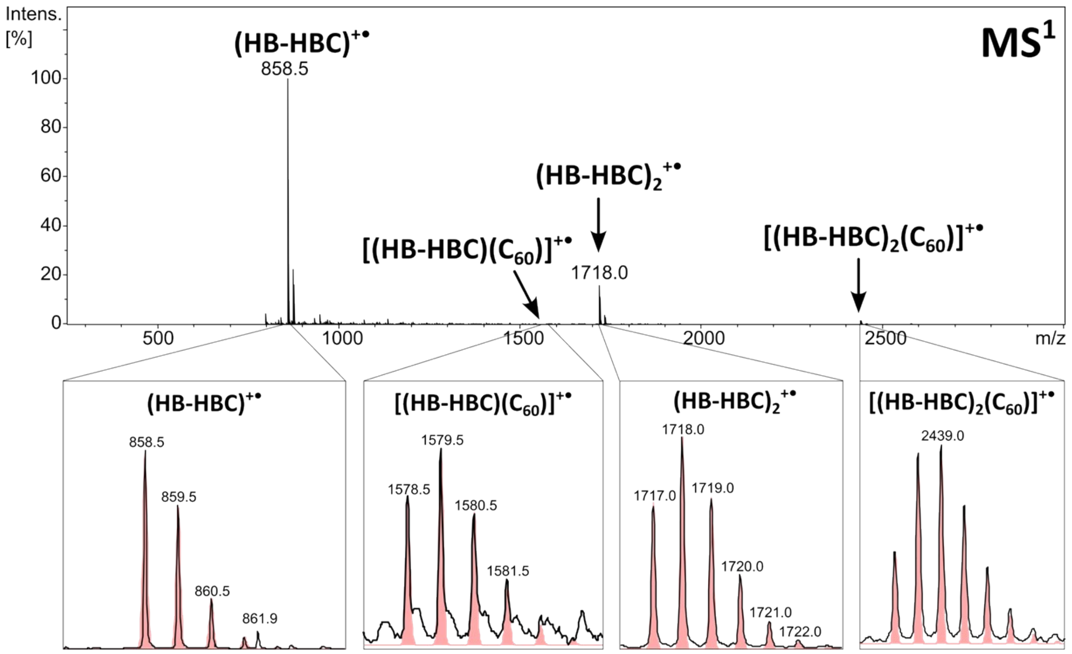

Figure S 3: MS[1] of the HB-HBC/C_{60} analyte solution. Only minor amounts of [1:1] and [2:1] HB-HBC fullerene complex was observed in the experiment.

2. Breakdown graph of $(HB\text{-}HBC)_2(C_{60})^+$

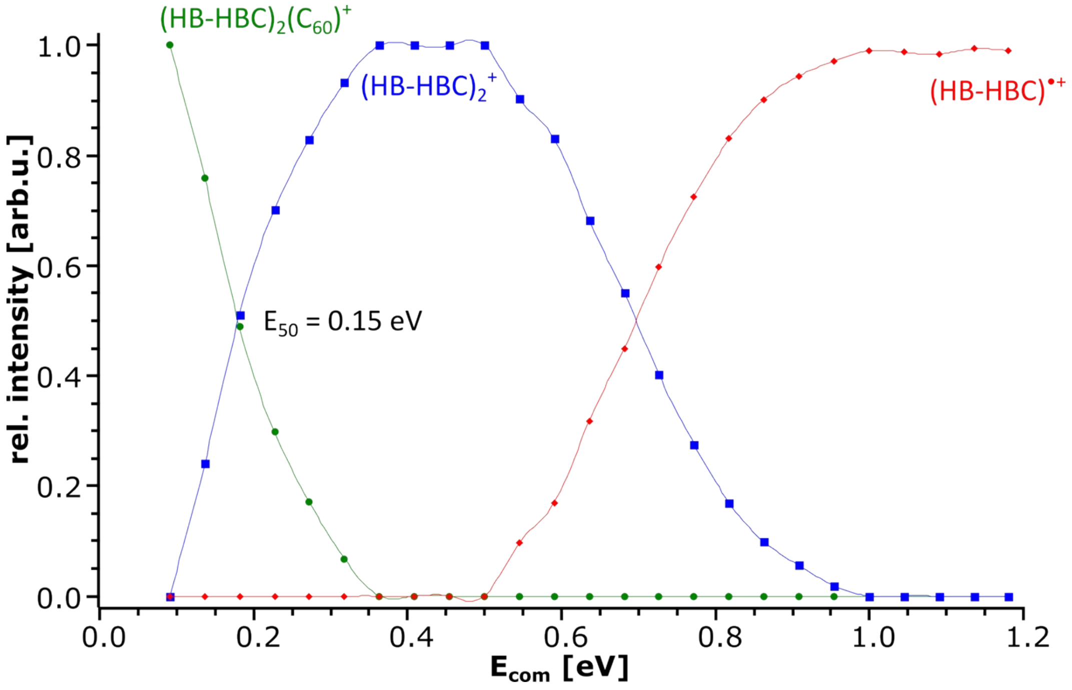

Figure S 4: Breakdown graph of the HB-HBC:C_{60} [2:1] complex, showing the loss of neutral C_{60} at low collision energy followed by the dissociation of the HB-HBC dimer at higher collision energy. This indicates that the C_{60} is only loosely bound to an HB-HBC dimeric structure.

3. MS^1 Spectra of NTH and C_{70}

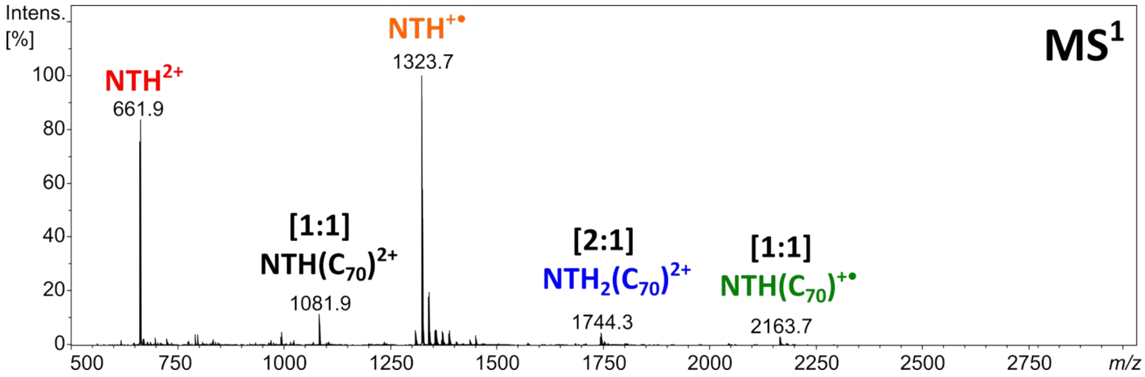

Figure S 5: MS^1 spectra of the NTH/C_{70} analyte solution.

4. Breakdown graphs of NTH(C_{70})

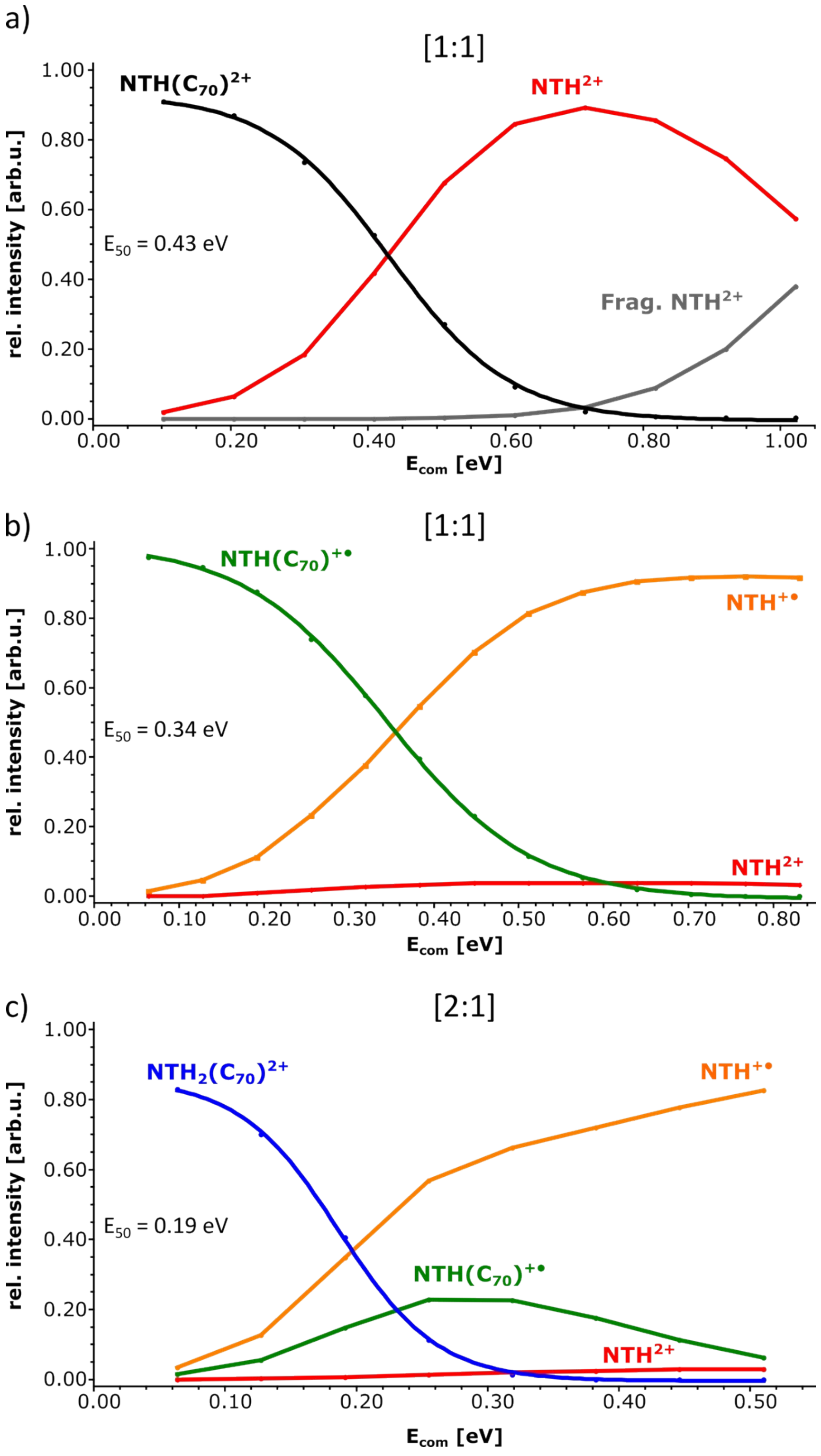

Figure S 6: Breakdown graphs of the selected NTH/C_{70} complexes.

5. MS[2] Spectra of $NTH(C_{78})^{2+}$

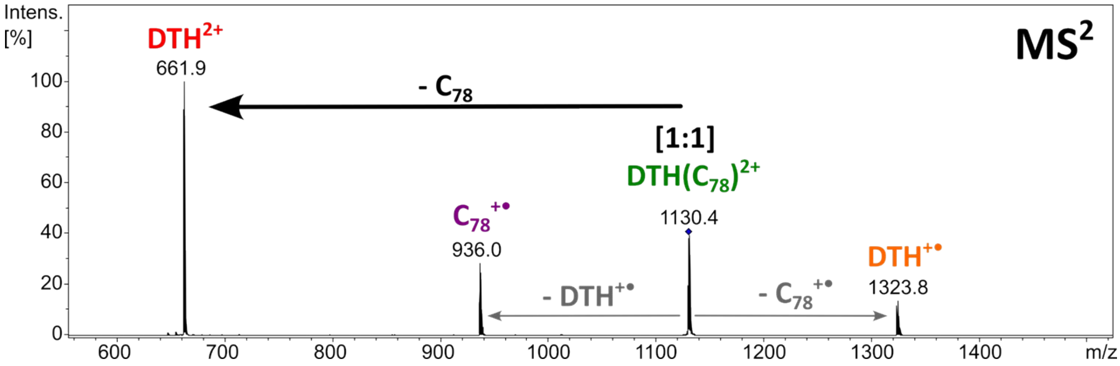

Figure S 7: MS^2 spectra of the doubly charged [1:1] complex of NTH and C_{78} (m/z 1130.4). Two fragmentation processes can be observed. The loss of neutral C_{78} (black) and the fragmentation into $C_{78}^{+\bullet}$ and $NTH^{+\bullet}$ (grey) indicate a charge transfer from the dicationic NTH to the fullerene.

6. E_{50} values of the NTH complexes with C_{60} and C_{70}

Table S 1: relative stability of the observed NTH/fullerene (C_{60} and C_{70}) complexes in eV

E_{50} in [eV]	**[1:1]** $NTH(C_{60/70})^{2+}$	**[1:1]** $NTH(C_{60/70})^{+}$	**[2:1]** $NTH_2(C_{60/70})^{2+}$	**[1:2]** $NTH(C_{60/70})_2^{+}$	**[2:3]** $NTH_2(C_{60/70})_3^{2+}$
**C_{60}**	0.31	0.28	0.15	0.18	0.18
**C_{70}**	0.43	0.34	0.19	---	---

7. DFT calculations: complex structures

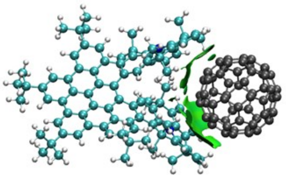

I-hta

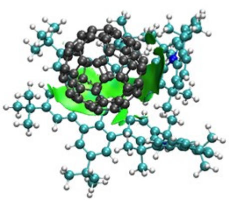

I -hbc-in

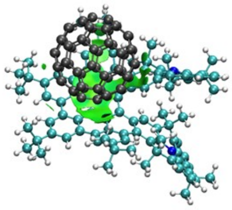

I -hbc-out

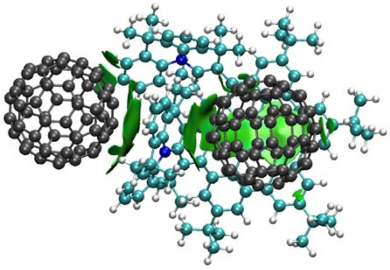

II -hta

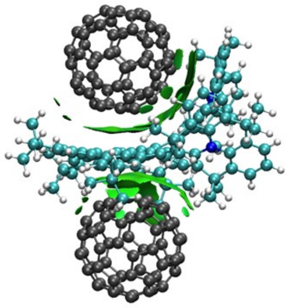

II -hbc

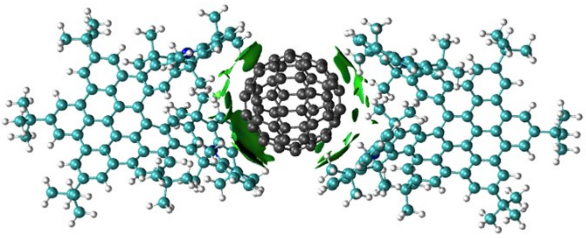

III-hta

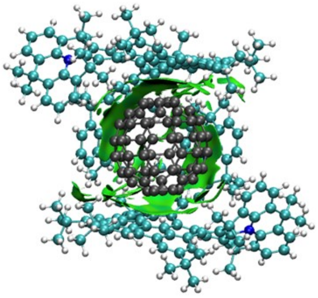

III -hbc

*Figure S 8: structures for the [1:1] (top), [1:2] (middle) and [2:1] (bottom) N*TH/C_{60} complexes.

8. Calculated fragmentation and ionization energies

Table S 2: Fragmentation energies of NTH/C_{60} [1:1] complexes in eV

**[1:1]** NTH / C_{60}	Neutral	Monocation		Dication	
	NTH/C_{60}	NTH^+/C_{60}	NTH/C_{60}^+	NTH^+/C_{60}^+	NTH^{2+}/C_{60}
I -hta	0.90	0.88	2.95	0.79	0.96
I -hbc-in	1.16	1.14	3.20	1.05	1.22
I-hbc-out	1.08	1.07	3.13	0.45	0.63

Table S 3: Fragmentation energies of NTH/C_{60} [2:1] complexes in eV

**[2:1]** NTH / C_{60}	Neutral	Monocation		Dication		
	NTH / NTH / C_{60}	NTH^+/ NTH / C_{60}	NTH / NTH / C_{60}^+	NTH^+/ NTH / C_{60}^+	NTH^+/ NTH^+/ C_{60}	NTH^{2+}/ NTH /C_{60}
II-hta	1.72	2.15	4.22	2.33	0.26	2.50
II-hbc	3.24	3.52	5.18	3.50	1.44	3.67

Table S 4: Fragmentation energies of NTH/C_{60} [1:2] complexes in eV

**[1:2]** NTH / C_{60}	Neutral	Monocation		Dication		
	NTH/ C_{60} / C_{60}	NTH^+/ C_{60} / C_{60}	NTH / C_{60}^+ / C_{60}	NTH^+/ C_{60}^+ / C_{60}	NTH^{2+}/ C_{60} / C_{60}	NTH/ C_{60}^+ / C_{60}^+
III-hta	1.97	1.91	3.97	1.30	1.47	3.36
III-hbc	2.20	2.18	4.24	1.67	1.84	3.73

We estimated the BSSE error for the fragmentation energies of the neutral DTH/C60 complex (Table S2, first column) using the counterpoise (CP) correction method. We obtain:
I-hta: CP correction = -0.05 eV
I-hbc-in: CP correction = -0.06 eV
I-hbc-out: CP correction = -0.05 eV
Thus, the CP correction yields a nearly uniform shift of the fragmentation energies, and the relative stabilities of the complexes are unaffected.

Table S 5: Calculated vertical ionization energies (PBE/def2-TZVP)

	**Ionisation energy**
NTH	^1IE = 5.33 eV
NTH	^2IE = 7.49 eV
C_{60}	^1IE = 7.32 eV
C_{70}	^1IE = 7.24 eV
C_{78} (D3)	^1IE = 6.63 eV
C_{78} (D3h)	^1IE = 6.93 eV
C_{78} (D2v)	^1IE = 6.88 eV

9. Energies and cartesian coordinates of the host-guest complexes

The calculated structures and energies (in Hartrees) can be found at:

https://doi.org/10.5281/zenodo.14534357

10. ^{1}H-, ^{13}C-NMR spectra and crystal structure of NTH

Nuclear Magnetic Resonance. ^{1}H- and ^{13}C-NMR spectra were recorded on a Bruker Avance 600 (Bruker, 601 MHz, for ^{1}H, 151 MHz for ^{13}C) spectrometer. Chemical shifts (δ) are reported in ppm and were referenced to the residual solvent signal as an internal reference ($(CD_3)_2CO$: 2.05 ppm for ^{1}H, 206.26 and 29.84 ppm for ^{13}C). Coupling constants (J) are given in Hz as observed and the apparent resonance multiplicity is reported as s (singlet), d (doublet), t (triplet), or m (multiplet). All signals of solvents and impurities are assigned according to literature.[1] All spectra were recorded at ambient probe temperature if not otherwise stated.

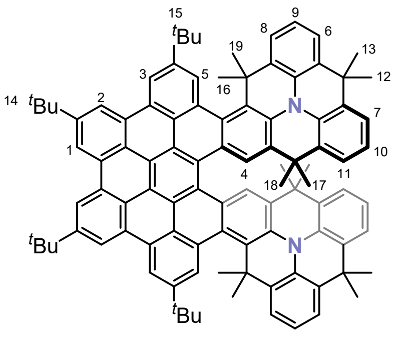

NTH

***N*-Heterotriangulene [5]helicene (NTH).**

^{1}H NMR (600 MHz, $(CD_3)_2CO$) *d* 9.38 (s, 2H, H^{1}), 9.33 (s, 2H, H^{2}), 9.24 (s, 2H, H^{3}), 8.82 (s, 2H, H^{4}), 8.30 (s, 2H, H^{5}), 7.78 (dd, J = 7.6, 1.2 Hz, 2H, H^{6}), 7.71 (td, J = 7.7, 1.3 Hz, 4H, H^{7}, H^{8}), 7.50 (t, J = 7.5 Hz, 2H, H^{9}), 7.30 (t, J = 7.5 Hz, 2H, H^{10}), 7.04 (dd, J = 7.5, 1.2 Hz, 2H, H^{11}), 1.91 (s, 6H, H^{12}), 1.88 (s, 6H, H^{13}), 1.82 (s, 18H, H^{14}), 1.72 (s, 18H, H^{15}), 1.66 (s, 6H, H^{16}), 1.33 (s, 6H, H^{17}), 1.03 (s, 6H, H^{18}), 0.78 (s, 6H, H^{19}) ppm.

^{13}C NMR (151 MHz, $(CD_3)_2CO$) *d* 150.6, 146.5, 141.3, 134.1, 132.3, 131.94, 131.86, 131.85, 131.7, 131.4, 131.1, 130.7, 129.6, 128.7, 128.4, 127.4, 126.8, 126.3, 126.0, 125.1, 125.0, 124.7, 124.1, 123.9, 123.6, 123.5, 123.1, 121.44, 121.36, 120.1, 120.5, 119.9, 119.7, 41.0, 36.5, 36.2, 36.1, 35.90, 35.87, 35.8, 34.3, 34.2, 32.3, 32.1, 30.4, 24.4 ppm.

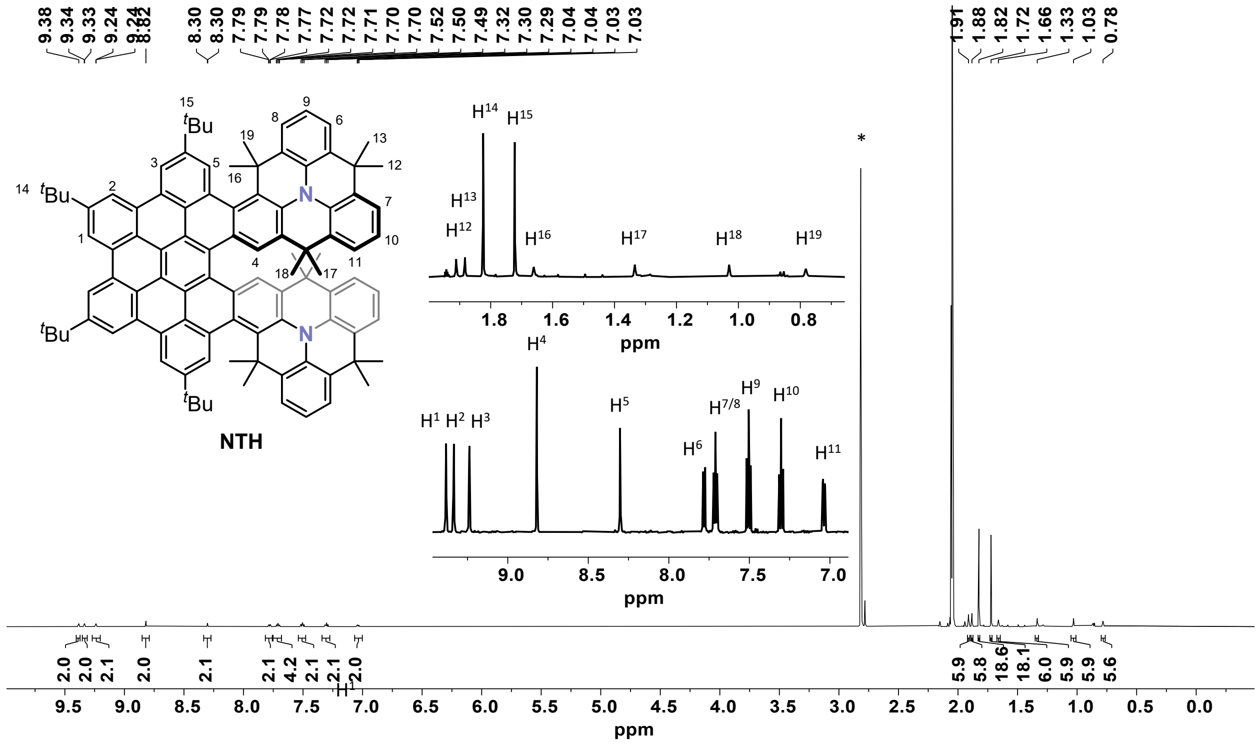

Figure S9:: ^{1}H *NMR spectrum (601 MHz,* $(CD_3)_2CO$*) of* ***NTH****; * H_2O.*

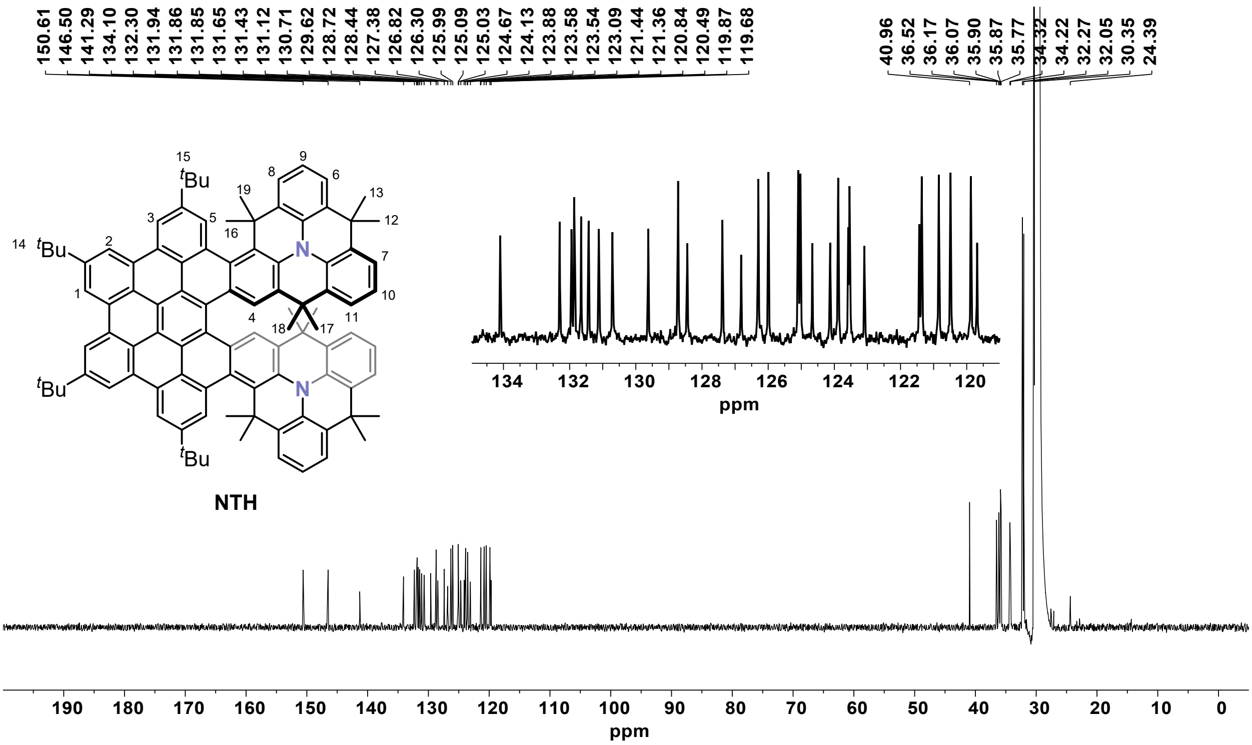

Figure S10: *^{13}C NMR spectrum (151 MHz, $(CD_3)_2CO$) of* ***NTH***.

[1] H. E. Gottlieb, V. Kotlyar, A. Nudelman, *J. Org. Chem.* **1997**, *62*, 7512.

Deposition number CCDC 2406553 contains the supplementary crystallographic data for **NTH**. The data can be obtained free of charge from The Cambridge Crystallographic Data Centre via www.ccdc.cam.ac.uk/structures.
